# Navigating Conflicting Rights Among Residents, Visitors, and Families in Long-Term Care Facilities

**DOI:** 10.1093/geroni/igaa057.065

**Published:** 2020-12-16

**Authors:** Angela Perone

**Affiliations:** University of Michigan, Ypsilanti, Michigan, United States

## Abstract

Background: An abundance of long-term care regulations creates a bevy of rights for nursing facility residents, staff, and families. Front-line workers and managers have significant discretion and responsibilities for interpreting these rights. Building on street-level-bureaucracy theory (Lipsky, 2010), which focuses on how front-line workers implement policy, this study examines how staff at various levels (direct care, mid-level professional, top management) resolve conflicting rights. Methods: This study employs a novel advanced multi-method qualitative design with semi-structured staff interviews (n=90), content analysis of long-term care facility policies (n=75), and participant observation of two facilities for a multi-layered comparative case study. Findings: Data analysis revealed variations in staff responses to conflicting rights regarding autonomy and safety (e.g. fall prevention, dementia, coronavirus) and discrimination (i.e. sexual/racial harassment). While harassment was rampant, direct care workers responded more deferentially to residents and often justified harassment as part of a customer service job in one’s home. Staff at all levels relied on teams to develop creative problem-solving approaches, but team composition and discretion varied significantly between facilities and staff levels. While staff included few social workers, staff heavily relied on them to adjudicate conflicting rights. Implications: Conflicting rights impact resident care and relationships among residents, staff, and families. This research provides policymakers and practitioners with new data about how staff resolve conflicting rights, which can facilitate stronger policies to support an overburdened and underpaid long-term care workforce. This research also expands street-level-bureaucracy theory to include managers and reveals how various team approaches can produce diverse solutions.

